# Advances in wound repair and regeneration: Systematic comparison of cell free fat extract and platelet rich plasma

**DOI:** 10.3389/fchem.2022.1089277

**Published:** 2022-12-21

**Authors:** Lifang Zhang, Chengjun Hu, Wenqing Xu, Dingyu Wu, Shaorong Lei

**Affiliations:** Department of Plastic Surgery, National Clinical Research Center for Geriatric Disorders, Xiangya Hospital, Central South University, Changsha, China

**Keywords:** PRP, CEFFE, skin wound repair, growth factor, regeneartive medicine

## Abstract

**Background:** Previous studies showed Cell free fat extract (CEFFE) and Platelet rich plasma (PRP) could effectively accelerate wound healing. However, the comparative study on curative effect is still lacking. A systematic comparison could provide more theoretical support and laboratory basis for the clinical application of CEFFE and PRP.

**Objective:** To compare the efficacy of CEFFE and PRP in promoting skin wound repair.

**Methods:** CEFFE and PRP were prepared according to the literature. The wound repair related factors were measured and compared. *In vitro*, the effects of both on cell migration, proliferation and tube formation were compared. *In vivo*, wound healing rate was measured on the 1st, 3rd, 9th, and 12th days after skin injury and treatment. Then the specimens were cut off for histological analysis.

**Results:** Although the total protein content of PRP was significantly around 19 times higher than that of CEFFE, there was no statistical difference in the content of BDNF, EGF and VEGF between CEFFE and PRP. Even the NT-3 content of CEFFE was just slightly higher than that of PRP. The concentration of b-FGF, HGF and TGF-β and PDGF-BB in PRP is higher than that in CEFFE, but there is only a very small difference between them. *In vitro*, PRP showed better efficacy than CEFFE in promoting fibroblast proliferation while there was no significant difference in promoting angiogenesis and fibroblast migration. Both PRP and CEFFE could significantly promote wound healing in mice. There was no statistical difference in wound healing between CEFFE and PRP groups *in vivo*. Immunohistochemical staining of Ki67&CD31 showed that there was no significant difference between PRP and CEFFE groups.

**Conclusion:** The effect of PRP and CEFFE in promoting wound healing was similar. In clinical practice, the acquisition of PRP is relatively more convenient. Containing no cells, CEFFE has the advantage of easier preservation. For patients who have discarded adipose tissue, or contraindications to PRP technology, CEFFE technology may provide a new option for skin wound repair.

## 1 Introduction

In recent years, the autologous extracts have shown great therapeutic potential in wound repair, such as CEFFE, PRP, etc. It has the following advantages: 1) Extracted from the own tissue of the human body, the autologous extracts, which have no ethical issues, avoid the risk of immune rejection and infection; 2) There are many kinds of growth factors in a high concentration, the proportion of each growth factor is consistent with the normal proportion in the body, and all the growth factors have the best synergistic effect (Chicharro-Alcantara et al., 2018; Yu et al., 2018).

In the process of natural wound healing, platelets first arrive at the wound and release α-particles, which are rich in a variety of growth factors, playing an important role in promoting wound healing (Nurden, 2018). Widely used in clinical practice, autologous platelet rich plasma (PRP) mimics and enhances this process. Platelet rich plasma (PRP) is an autologous blood derived product extracted by centrifugation from whole blood. PRP contains a variety of growth factors, including platelet derived growth factor (PDGF), basic fibroblast growth factor (b-FGF), vascular endothelial growth factor (VEGF) and transforming growth factor-β (TGF-β) and other substances involved in promoting tissue regeneration and repair (Salamanna et al., 2015). The synergistic effect of these factors could stimulate the cell process, for example, by inducing the migration, proliferation, differentiation and stability of endothelial cells in new blood vessels to accelerate the blood circulation reconstruction and collagen deposition of damaged tissues (Xu et al., 2020); The migration, proliferation and activation of fibroblasts could repair damaged connective tissue, promote fibrosis, restore damaged extracellular matrix, and accelerate skin wound repair (Law et al., 2017). Many studies have confirmed the important role of PRP in tissue regeneration and wound healing (Salamanna et al., 2015). Because of its simple preparation, high growth factor content and low immunogenicity, PRP has been widely used in clinical treatment.

Cell-free fat extract (CEFFE) is a new kind of autologous extract since 2018. The preparation process of CEFFE is roughly as follows: use mechanical methods to break cells in nano fat, and then remove cell components, lipid residues, oil droplets, cell fragments and extracellular matrix. The purified liquid part is the “cell-free fat extract” (CEFFE) (Yu et al., 2018). Previous studies have confirmed that it contains a large number of growth factors to promote regeneration and repair, which could partially simulate the role of paracrine process of stem cells in tissue defect repair (Yu et al., 2018). Research shows that CEFFE contains a variety of growth factors, including brain-derived neurotrophic factor (BDNF) and transforming growth factor-β (TGF-β), Hepatocyte growth factor (HGF), basic fibroblast growth factor (b-FGF), vascular endothelial growth factor (VEGF), platelet derived growth factor (PDGF), epidermal growth factor (EGF), neurotrophic factor (NT-3), and other substances involving in promoting tissue regeneration and repair. CEFFE could also induce the migration, proliferation, differentiation and stability of endothelial cells in new blood vessels, and also promote the migration, proliferation and activation of fibroblasts (Zheng et al., 2019; Deng et al., 2020). Current research shows that it could accelerate wound healing as effectively as PRP.

**FIGURE 1:**
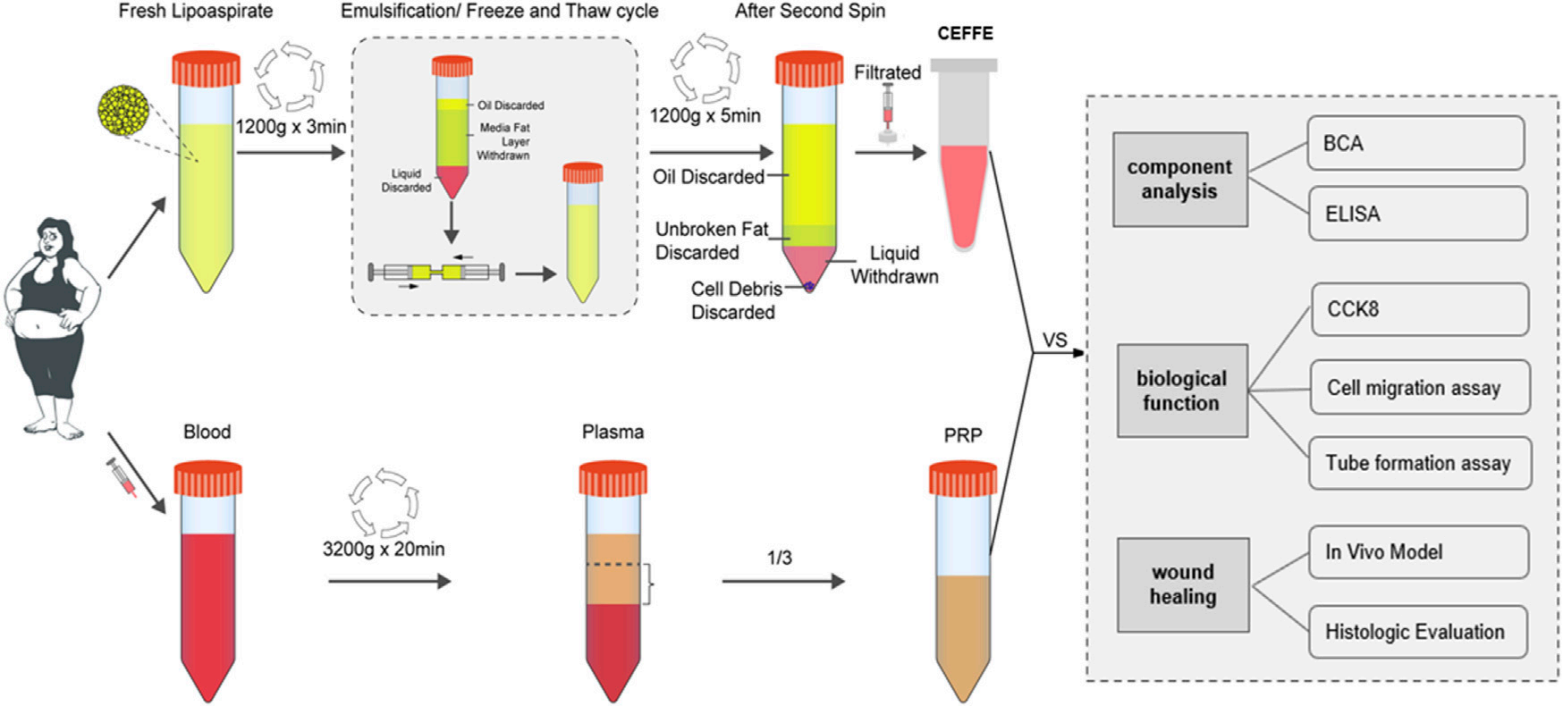
Flow chart of the comparison of CEFFE & PRP.

However, the comparative study of CEFFE and PRP in the treatment of wound defects is still lacking. Therefore, in this study, we systematically compared CEFFE and PRP in wound related factors, cell experiments and skin wound repair models, in order to provide more theoretical support and laboratory basis for the clinical application (Figure 1).

## 2 Materials and methods

### 2.1 Preparation of CEFFE and PRP

The remaining blood of human adipose tissue and preoperative blood examination was obtained from the healthy people who underwent liposuction for weight loss. Written informed consent was obtained from each donor.

Centrifuge the obtained fat at 1200 g rotating speed for 3 min. Suck out the upper oil layer and the lower fluid layer, and take out the middle fat layer for mechanical emulsification. Emulsification is achieved by pushing the fat back and forth 30 times between two 20 ml syringes. The emulsified fat was frozen at −80°C for 24 h and then thawed at 37°C to further destroy cells in adipose tissue. After the thawing, centrifuge the emulsified fat at 1200 g speed for 5 min, and then discard the upper oil, fat layer and debris, carefully suck out the third layer of water. Finally, filter it with .22 µm filter, and then freeze it at −20°C for later experimental purposes.

According to the manufacturer’s instructions, the disposable human vein blood sample collection container (Liuyang Sanli Medical Technology Co., Ltd., China) was used to collect blood. The blood was centrifuged at 3,200 rpm for 20 min by one-step centrifugation. The blood was divided into three layers, and the lower 1/3 of the upper clear liquid was taken as PRP. Frozen it at −20°C for later experimental purposes. The study was approved by the Ethics Committee of Xiangya Hospital, Central South University of China. (No. 202209604).

### 2.2 BCA and ELISA

According to the manufacturer’s instructions, the total protein concentration of CEFFE and PRP was measured with BCA protein determination kit. The total protein content was quantified as a standard and measured at 562 nm using a microplate reader. Double antibody sandwich enzyme-linked immunosorbent assay (ELISA) was used to quantitatively detect the levels of various factors (BDNF, b-FGF, HGF, EGF, NT-3, TGF-β, PDGF-BB, and VEGF) in CEFFE and PRP.

### 2.3 Cell culture

Human fibroblasts and human umbilical vein endothelial cells (HUVECs) (from the central laboratory of Xiangya Hospital of Central South University) were cultured in DMEM high glucose medium (Gibco) containing 10% FBS (fetal bovine serum), with an additional addition of 1% Penicillin Streptomycin (Gibco). The medium was changed every 2–3 days, and cultured at 37°C, 5% CO_2_ and 95% air in a humid atmosphere.

### 2.4 CCK-8

Use the cell counting kit-8 (CCK-8) to detect cell proliferation, and 3×10^3^ cells per hole were inoculated with fibroblasts in 96 well plates. In DMEM medium containing 10% FBS, add 10 μl CEFFE, PRP or PBS. Observe the cell proliferation on the 1st, 3rd, 5th days, and record the absorption spectrum at 450 nm with the microplate reader. The data is expressed as a ratio of OD, relative to the value of the control group.

### 2.5 Cell migration

Inoculate fibroblasts and HUVECs into 6-well plates (1 × 10^4^ cells for each well) and wait for them to grow into monolayer. Evenly draw a horizontal line to remove the cells. Add DMEM medium, and then add 10% PRP or CEFFE or PBS to the medium. It was cultured in a humid atmosphere of 37°C, 5% CO2 and 95% air. Use an optical microscope to take pictures at 0 h and 24 h, and then use ImageJ software to analyze the data. The data is expressed as the relative percentage of migration = [(A0−At)/A0] × 100%, where A0 is the initial wound area (*t* = 0) and At is the wound area at 0 h and 24 h.

### 2.6 Tube forming

Add cell suspension on 96 well plates coated with 70μl Matrigel glue (Corning, United States), 5 × 10^3^ HUVECs/100ul per well, and then add 10ul CEFFE, PRP or PBS respectively to the culture medium. Incubate the plates at 37°C and 5% CO_2_ for 8 h. The tubule formation was photographed under an optical microscope, and the number of formed connections was calculated using ImageJ software (NIH).

### 2.7 Skin wound repair

The BALB/C mice (8 weeks, 36–40 g) were divided into three groups: PRP, CEFFE, and PBS groups. All animal experiments were conducted in accordance with the National Standards for Laboratory Animal Environment and Facilities and the Guide for Raising and Using Laboratory Animals of Central South University. All mice were given general anesthesia. Shave off the hair on the back area, and use scissors to create a round full-thickness wound with a diameter of 10 mm in the central area of the back of the mouse. Inject PRP, CEFFE or PBS 200 μl subcutaneously on the wound for five consecutive days. Digital images of the wounds were captured on the 1st, 3rd, 9th, and 12th days. Use Image Pro Plus software version 6.0 to analyze the image. Wound closure is expressed as a percentage of the original wound area. All mice were euthanized at the 12th day.

### 2.8 Histological staining

The specimens were fixed with 4% paraformaldehyde and embedded in paraffin. Then the specimens were sectioned and stained with hematoxylin eosin (H&E) and Masson to observe the tissue morphology and collagen formation. CD31 immunohistochemical staining sections were used to observe the angiogenesis, and Ki67 immunohistochemical staining specimens were used to observe the number of cells in the proliferation phase.

### 2.9 Statistical analysis

GraphPad statistical software was used for statistical analysis. The mean ± standard deviation (SD) was used to express the experimental data of each group. Single factor analysis of variance (ANOVA) or non-parametric test were used to analyze the differences between groups. The significance level was set as **p < .05.

## 3 Results

### 3.1 Preparation and identification of CEFFE and PRP

#### 3.1.1 Preparation of CEFFE and PRP

**FIGURE 2:**
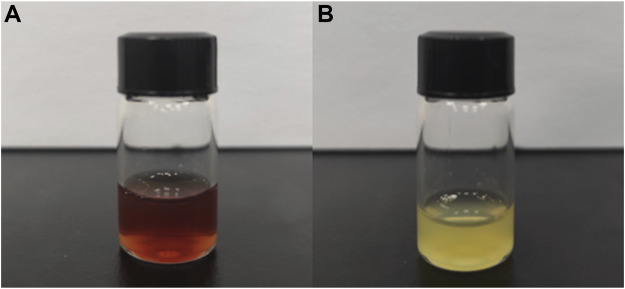
Preparation of CEFFE&PRP. **(A)**: CEFFE, light red; **(B)**: PRP, light yellow.

Shown in Figure 2A, the newly extracted CEFFE is a light red liquid substance. And shown in Figure 2B, the newly extracted PRP is a light-yellow liquid substance.

#### 3.1.2 BCA and ELISA

**FIGURE 3:**
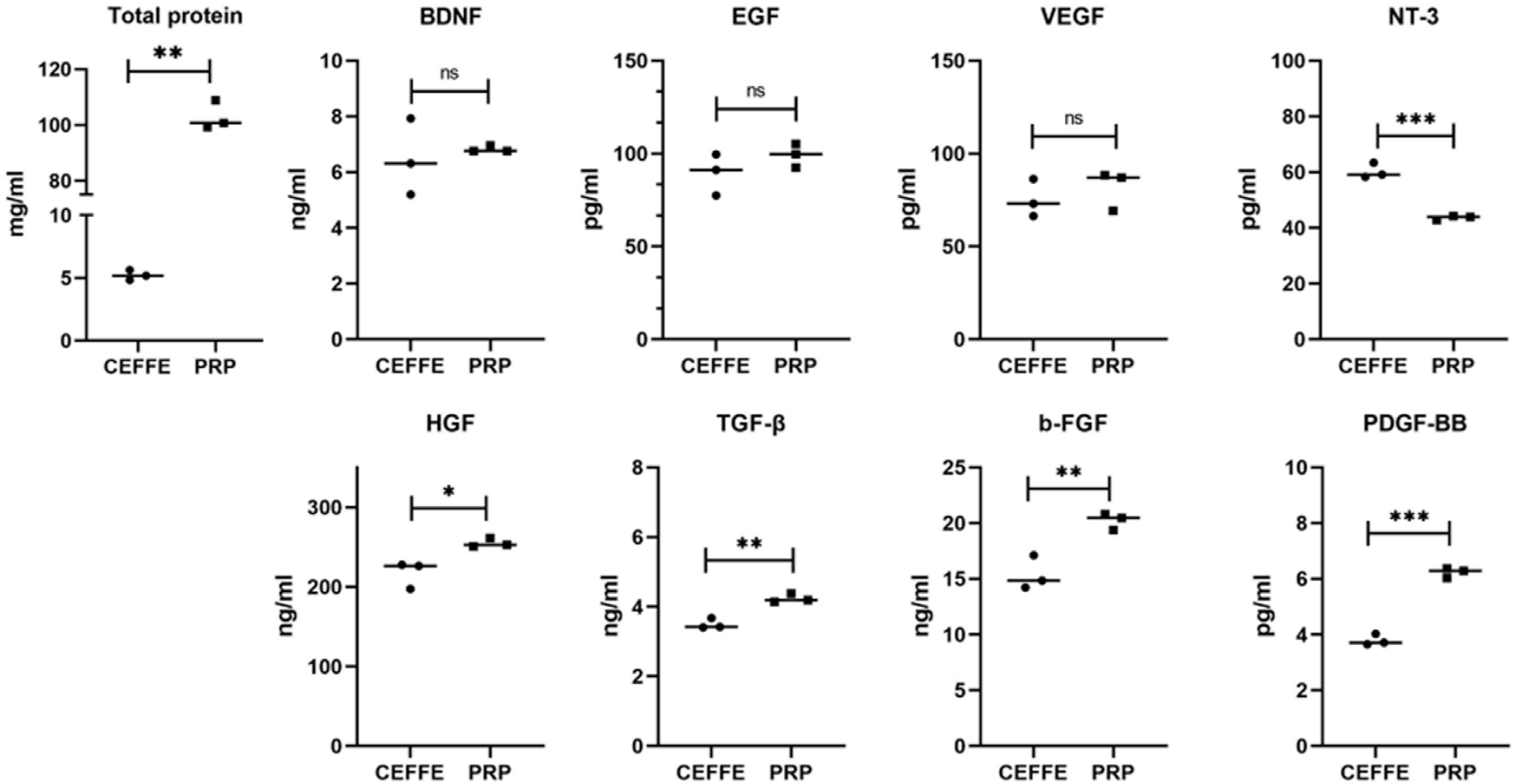
BCA and ELISA. The total protein concentration was measured by BCA in PRP and CEFFE, and the concentration of various factors (BDNF, EGF, VEGF, NT-3, HGF, TGF-β, b-FGF, and PDGF-BB) was measured by ELISA in PRP and CEFFE. *n* = 3, *p < .05, **p < .01, ***p < .001.

The average total protein concentration of CEFFE samples is 5.217 ± 401 mg/ml, ranging from 4.835 mg/ml to 5.635 mg/ml. The average protein concentration of PRP samples is 102.9 ± 5.196 mg/ml, ranging from 99.17 to 108.9 mg/ml, which is generally 19 times higher than that of CEFFE. Figure 3 shows the total protein concentration obtained from CEFFE and PRP samples.

The growth factors in CEFFE and PRP of each sample were measured by ELISA. As shown in Figure 3, there is no statistical difference in the contents of BDNF (CEFFE: 6.479 ± 1.375 ng/ml, PRP: 6.826 ± 0.112 ng/ml), EGF (CEFFE: 89.44 ± 11.17 pg/ml, PRP: 99.08 ± 6.377 pg/ml) and VEGF (CEFFE: 75.27 ± 10.16 pg/ml, PRP: 81.55 ± 10.61 pg/ml); The content of NT-3 (60.25 ± 2.703 pg/ml) in CEFFE is 1.37 times that in PRP (43.71 ± 0.737 pg/ml); The content of b-FGF (20.22 ± 0.744 ng/ml) in PRP was 1.31 times that in CEFFE (15.4 ± 1.519 ng/ml); The content of HGF (254.8 ± 5.459 ng/ml) in PRP was 1.17 times that in CEFFE (217 ± 17.05 ng/ml); The content of TGF-β (4.236 ± 0.122 ng/ml) in PRP is 1.21 times that in CEFFE (3.503 ± 0.147 ng/ml); The content of PDGF-BB (6.229 ± 0.177 pg/ml) in PRP is 1.64 times that in CEFFE (3.796 ± 0.201 pg/ml) (*p* < 0.05). Although the total protein content of PRP was significantly higher than that of CEFFE (about 19 times), there was no statistical difference in the content of BDNF, EGF, and VEGF between CEFFE and PRP, and even the NT-3 content of CEFFE was slightly higher than that of PRP. The concentration of b-FGF, HGF and TGF-β and PDGF-BB is higher than that of CEFFE, but there is no significant multiple difference between them.

#### 3.1.3 Promoting proliferation

**FIGURE 4:**
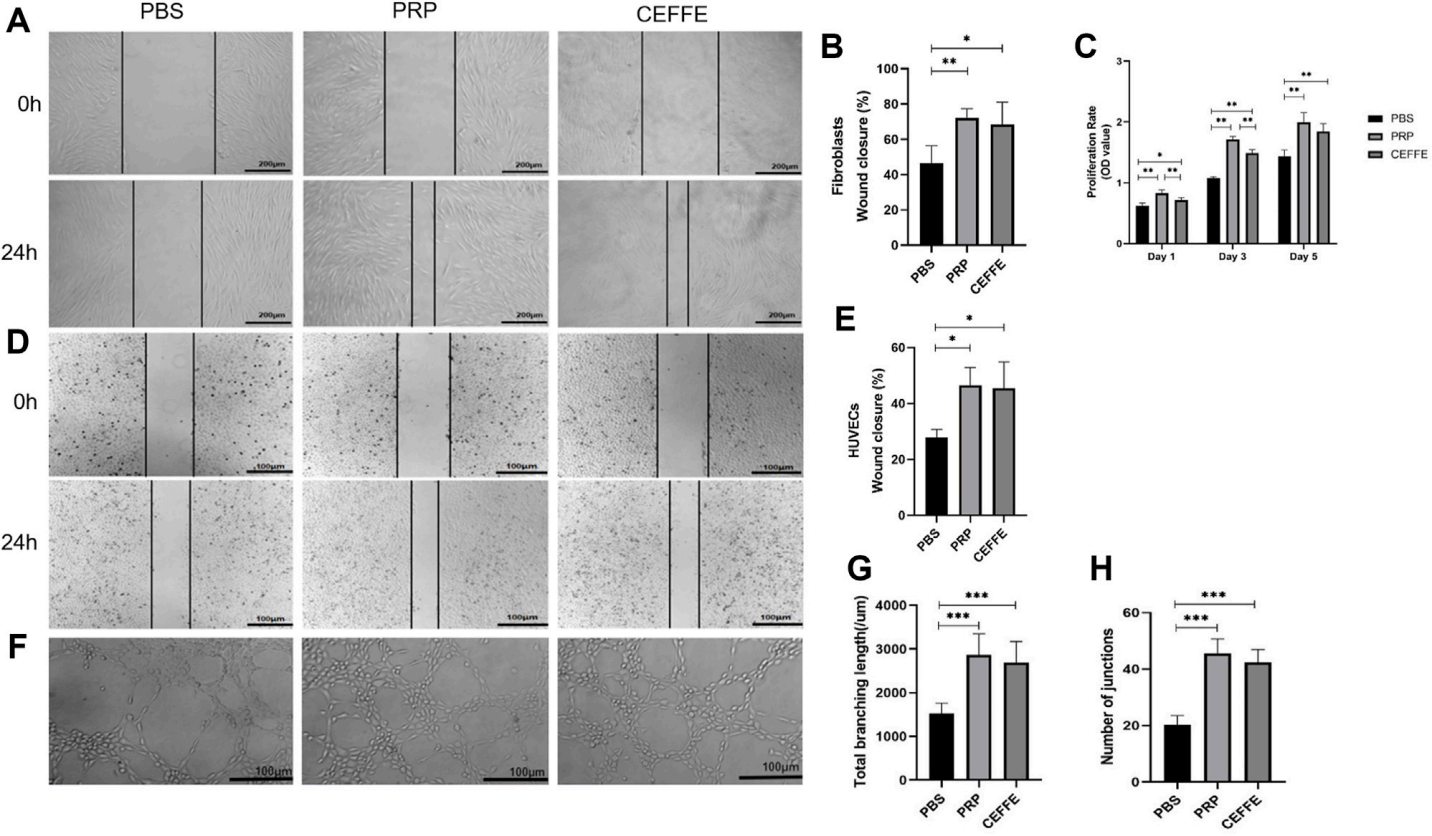
Migration, proliferation and tube formation **(A)** Promoting the migration of fibroblasts by PRP and CEFFE. **(B)** The percentage of scar closure of fibroblasts was quantified after 24 h. **(C)** The proliferation curve of fibroblasts was determined by CCK-8 during 5 days of culture. **(D)** Promoting the migration of HUVECs by PRP&CEFFE. **(E)** The percentage of scar closure of HUVECs was quantified after 24 h. **(F)** Comparison of tube forming. **(G)** Quantification of the head pipe length. **(H)**: Evaluation of the number of branch points in each group (/mm2). A Scale is 200 μm. D&F scale is 100 μm. *n* = 3, *p < .05, **p < .01, ***p < .001.

As shown in Figure 4C, on the 1st and 3rd day, the cell proliferation rate of the three groups began to show difference. The proliferation efficiency of PRP group was the best, followed by CEFFE group, both of which were better than PBS group. Moreover, on the 5th day, the proliferation of fibroblasts in PRP and CEFFE groups were significantly increased compared with the PBS group, and there was no significant difference between PRP and CEFFE groups.

#### 3.1.4 Promoting migration

As shown in Figures 4A, B, D, E, compared with the PBS group, PRP and CEFFE groups could promote the migration of fibroblasts and HUVECs greatly. There is no significant difference between PRP and CEFFE groups.

#### 3.1.5 Tube forming

Compared with PBS group, more tubular structures were observed in PRP and CEFFE groups (Figure 4F). It was confirmed by calculating the number of branch points and measuring the total length of the three groups (Figures 4G, H). There is no significant difference between PRP and CEFFE groups.

### 3.2 Comparison of skin wound repair

#### 3.2.1 Change of skin wound area

**FIGURE 5:**
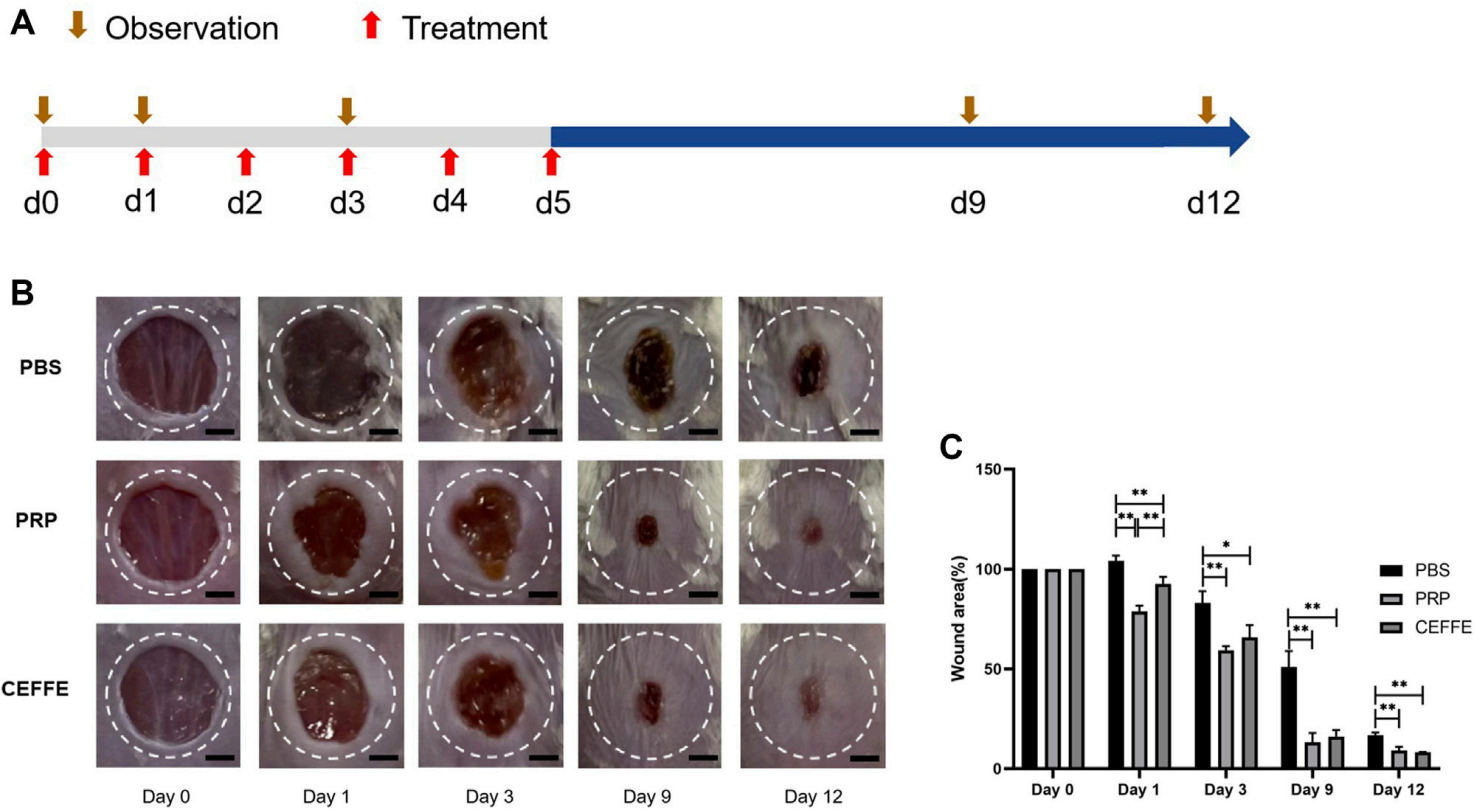
Comparison of wound repair **(A)** Flowchart. **(B)** The images of wound healing on days 0, 1, 3, 9 and 12. **(C)** The percentage of wound area in each group at 0, 1, 3, 9, 12 days after injury. Quantitative analysis of wound closure showed that the healing rate of CEFFE and PRP groups was higher than that of the control group. There was no significant difference between CEFFE and PRP groups. Ruler = 2 mm. *n* = 3, *p < 0.05, **p < 0.01.

Compared with the control group, the wound area of CEFFE and PRP groups decreased from the 1st day. On the 9th and 12th days, the wound size of CEFFE and PRP groups decreased significantly faster than that of the control group. There was no significant difference between CEFFE and PRP groups (Figure 5).

#### 3.2.2 HE and Masson

**FIGURE 6:**
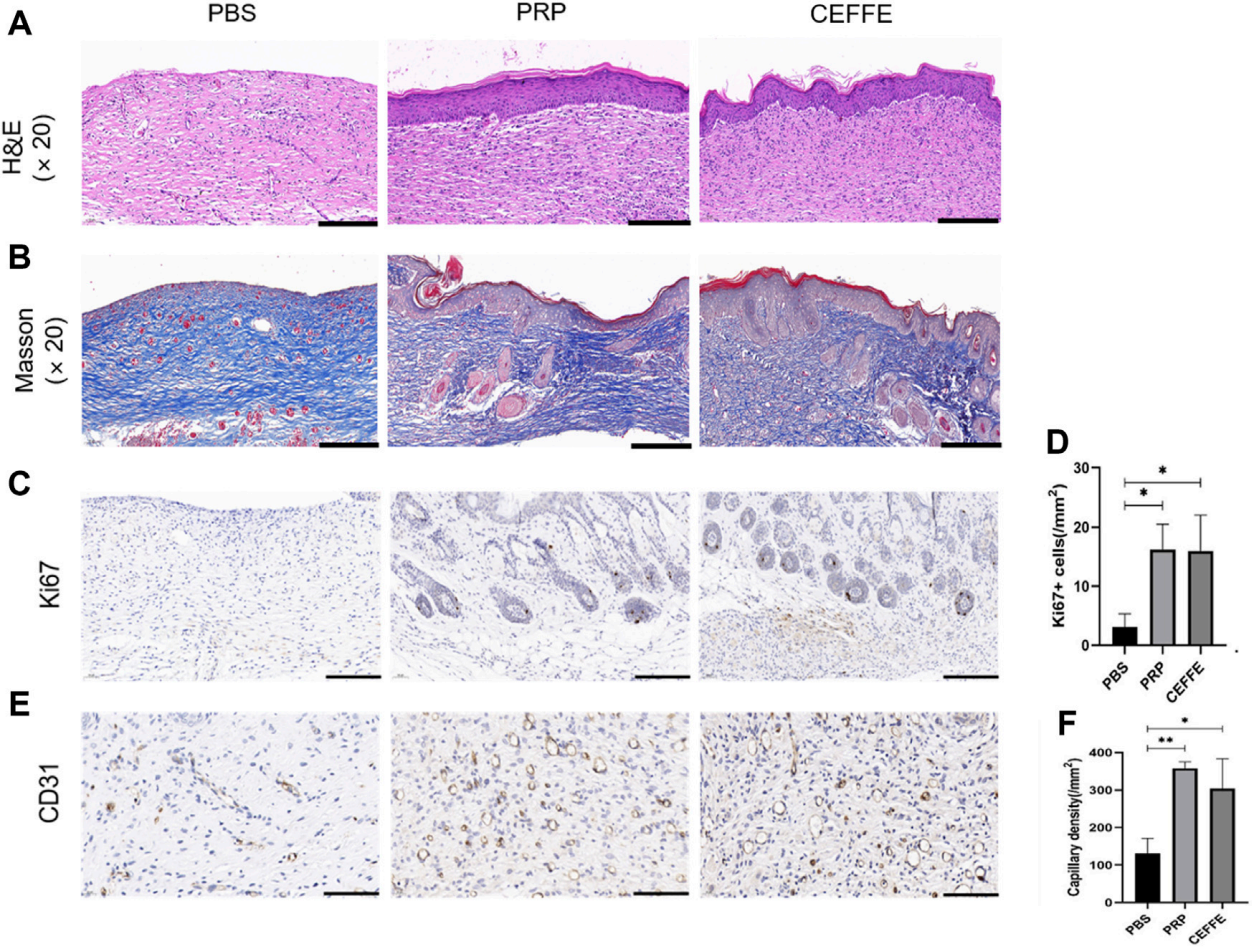
Comparison of histological staining **(A)** HE staining of skin wound in each group. **(B)** Masson staining of skin wound in each group. **(C,D)**: Ki67 staining image and quantitative analysis. **(E,F)**: CD31 staining image and quantitative analysis. **(A,B)** Scale is 100 μ m; **(C,E)** Scale is 200 μm. *n* = 3, *p < 0.05, **p < 0.01.

HE staining showed that the wounds in CEFFE and PRP groups showed complete and continuous re epithelization; There was almost no re epithelization in the control group. Masson staining showed that the collagen bundles in PBS group were sparse, while the collagen fibers (dyed blue) in PRP and CEFFE groups were significantly increased, and the collagen fibers were arranged orderly. There was no significant difference between PRP and CEFFE groups (Figure 6B).

#### 3.2.3 Immunohistochemical staining of Ki67 and CD31

The expression of Ki67 in regenerated tissue was detected to evaluate the proliferation activity of dermal and epidermal cells in skin wounds. As shown in Figures 6C, D, the number of proliferating cells in PRP and CEFFE groups was significantly higher than that in the control group (*p* < 0.05). There was no significant difference between PRP and CEFFE groups (Figures 6C, D).

The expression of CD31microvessels in regenerated tissue were detected to evaluate the angiogenesis promoting ability of CEFFE and PRP *in vivo*. As shown in Figures 6E, F, many newly formed and mature blood vessels can be seen in CEFFE and PRP groups, and their number and density are significantly higher than those in the control group. There was no significant difference between PRP and CEFFE groups (Figures 6E, F).

## 4 Discussion

In the process of natural wound healing, platelets first arrive at the wound site and react, playing a key role in the initiation of wound healing (Nurden, 2018). Platelet release α-granules, including coagulation factor, fibrinogen, platelet thromboplastin, platelet reactive protein, PDGF, TGF-β, VEGF, EGF, IGF, calcium, 5-hydroxytryptamine, histamine, and hydrolase, etc (Schar et al., 2015). These factors play an important role in wound healing. Therefore, the application of autologous platelet rich plasma (PRP) at the wound site is considered to be a new strategy that could promote wound healing and tissue regeneration (Xu et al., 2020). Stem cells have obvious effects on repairing tissue defects. Paracrine function may play a more critical role in the process of stem cells repairing tissue defects. It has been proved that CEFFE contains a large number of growth factors and shows the characteristics similar to stem cells in promoting tissue regeneration and repair (Zheng et al., 2019).

Both PRP and CEFFE are autologous extracts, so there is no risk of immune rejection and infection, nor ethical risk, and they are safer and more reliable than the current commercial growth factors. Both of them contain a variety of high concentration growth factors, which could promote regeneration and repair tissue. The proportion of each factor is close to that under natural conditions, which is more synergistic than using a single growth factor.

Both PRP and CEFFE are extracted from human tissue. PRP comes from blood, while CEFFE comes from adipose tissue. Prepared of centrifugation only, PRP contains platelet and other cells. Prepared by mechanical emulsification, freezing and centrifugation, CEFFE contains no cells. Interestingly, although coming from blood, PRP is light-yellow liquid; and although coming from fat, CEFFE appears as light-red liquid (Figure 2). PRP is yellow, mainly because centrifugation removes red blood cells from the blood. However, there is no research to reveal the reason why CEFFE appears light red. Some studies have shown that carotenoids are widely present in fat, so we believe that they may be closely related to the reddish color of CEFFE (Bonet et al., 2016).

**FIGURE 7:**
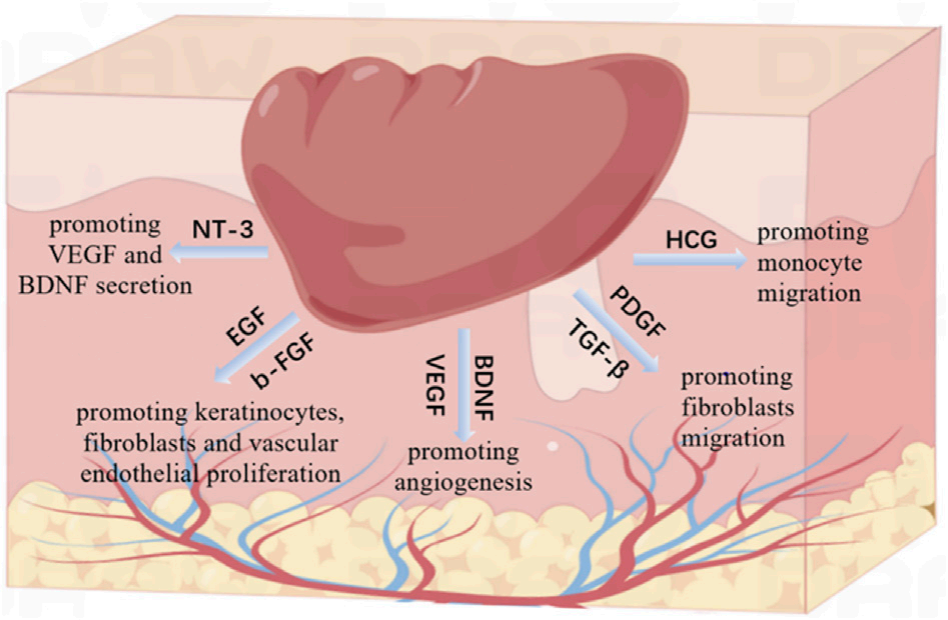
The molecular mechanisms of the main related growth factors contained in CEFFE and PRP.

Wound healing is an orderly process, including inflammation, proliferation (epithelization, angiogenesis) and remodeling (collagen deposition and scar tissue formation) (Dolati et al., 2020). This process is involved by various growth factors and precisely controlled by complex interactions between them. In this study, we identified the main related growth factors of PRP and CEFFE through ELISA (Figure 3). We found that both PRP and CEFFE contain rich growth factors just as the previous studies reported (Salamanna et al., 2015; Yu et al., 2018). And the molecular mechanisms of these growth factors have already been extensively studied (Figure 7). In the inflammatory stage, HGF is an inflammatory factor that plays a role by promoting monocyte migration, inflammatory cytokine release, and adult monocyte antigen presentation (Saghizadeh et al., 2005). In the early stage of tissue repair, PDGF and TGF- β could chemically attract fibroblasts into the wound area (Wahl et al., 1989). They play a key role in the activation of mesenchymal cells and fibroblasts, as well as the recruitment and activation of neutrophils and macrophages (Larouche et al., 2018). In the middle and late stage, BDNF, NT-3, EGF, VEGF, and b-FGF began to play a major role. Among them, BDNF and NT-3 are both nerve growth factor families, which play an important role in cardiovascular development at an early stage, and also have the ability to promote angiogenesis (Shen et al., 2013). EGF can stimulate the proliferation of keratinocytes, fibroblasts and vascular endothelial cells and enhance the production of fibronectin (Tiaka et al., 2012; Gainza et al., 2015). VEGF can stimulate angiogenesis and promote endothelial cell migration and proliferation (Werner and Grose, 2003; Lee et al., 2011; Losi et al., 2013). Studies have shown that NT-3 can significantly promote the secretion of VEGF and BDNF in MSCs, and accelerate the wound healing of diabetic feet and other ischemic ulcers. Epidermal growth factor (EGF) and basic fibroblast growth factor (b-FGF) can promote the proliferation of fibroblasts, so they could be used to treat diabetic foot (Blakytny and Jude, 2009). Thus, we did not repeat the verification work in molecular mechanisms. CEFFE and PRP have been proved to have good effects in the field of wound repair, but there is no research comparing the main growth factors of the two. We compared the total protein content and the main related growth factors of them. Results showed that although the total protein content of PRP was significantly higher than that of CEFFE (about 19 times), there was no statistical difference in the content of BDNF, EGF and VEGF between the two, and even the NT-3 content of CEFFE was slightly higher than that of PRP. Although the concentration of b-FGF, HGF and TGF-β, and PDGF-BB in PRP is higher than that in CEFFE, there is no significant multiple difference between them. Therefore, we believe that both of them have a good ability to accelerate wound healing, while PRP is slightly higher than CEFFE in the content of some inflammatory factors.

PRP is slightly stronger than CEFFE in promoting fibroblast proliferation on 1st, 3rd day (as shown in Figure 4), which is consistent with the conclusion that the b-FGF and other factors in PRP is slightly higher than that in CEFFE. However, there was no significant difference between PRP and CEFFE in promoting the migration of fibroblasts and HUVECs. And there was no significant difference between PRP and CEFFE in promoting the tube formation.

Comparing the effect in promoting wound healing, we found that the speed of the CEFFE and PRP groups was significantly faster than that of the control group (Figure 5). The wound healing speed of PRP group was slightly faster than that of CEFFE group on the first day, but after 3 days there was no significant difference between the two groups. HE staining showed that no continuous epidermal formation was observed on the unhealed wound in PBS group, while the regenerative epithelium of PRP and CEFFE groups was continuous and complete, and the thickness of regenerative epithelium was significantly higher than that of PBS group (Figure 6). Masson staining showed the same: the collagen bundles in PBS group were sparse, while in PRP and CEFFE groups the deposition of collagen fibers (collagen fibers dyed blue) was significantly increased, and the collagen fibers were arranged orderly (Figure 6). There is no obvious difference between the PRP and CEFFE groups in HE and Masson staining. Ki67 staining showed that the number of cells in the proliferation phase in the two groups was significantly higher than that in the control group, but there was no statistical difference between PRP and CEFFE groups. CD31 staining showed that the density of new capillaries in PRP and CEFFE groups was significantly higher than that in PBS group, and there was no significant difference between PRP and CEFFE groups. From the above analysis, the difference in the content of factors between PRP and CEFFE could not enough lead to the difference in promoting wound repair. Combined with ELISA and *in vitro* experimental data, we believe that there are two possible mechanisms: 1) Both PRP and CEFFE can provide blood supply reconstruction for injured tissues by inducing the migration, differentiation and stability of endothelial cells in new blood vessels; 2) Both of them could enhance dermal collagen secretion to repair damaged tissue by promoting fibroblast activation.

However, their sources and extraction processes of PRP and CEFFE are completely different, which determines that they would have different advantages. Direct extraction from patient blood, the biggest advantage of PRP is that the acquisition of PRP is relatively more convenient. And PRP contains a large amount of fibrin, which could provide a good scaffold for repairing related cells, shrink the wound and promote blood coagulation. Furthermore, PRP can be coagulated with thrombin into a gel, which can not only glue the tissue defects, but also prevent the loss of platelets, so that the platelets can secrete growth factors in the local area for a long time. Extracted from adipose tissue and containing no cells, CEFFE also has advantages: 1) for patients with contraindications to PRP technology, such as platelet dysfunction and severe anemia (Kobayashi et al., 2016), CEFFE technology may provide a new choice for wound repair; 2) CEFFE is suitable for long-term cryopreservation because it does not contain cells (Yu et al., 2018). Therefore, for patients who need liposuction or fat removal, the remaining fat could be used to extract CEFFE, which could be stored for wound repair and other long-term treatment.

## 5 Summary

In this study, we compared CEFFE and PRP in promoting wound healing. *In vitro*, PRP showed stronger ability than CEFFE in promoting fibroblast proliferation, and there was no significant difference in promoting angiogenesis and fibroblast migration. Also, there was no significant difference between PRP and CEFFE in promoting wound healing *in vivo*. The acquisition of PRP is relatively more convenient. Containing no cells, CEFFE has the advantage of easier preservation. Although the application of CEFFE has some advantages over PRP, CEFFE still cannot completely replace PRP for wound treatment. For patients who have discarded adipose tissue, or contraindications to PRP technology, CEFFE technology may provide a new choice for wound repair.

## Data Availability

The original contributions presented in the study are included in the article/supplementary material, further inquiries can be directed to the corresponding authors.
